# Association of Circulating Platelet Extracellular Vesicles and Pulse Wave Velocity with Cardiovascular Risk Estimation

**DOI:** 10.3390/ijms231810524

**Published:** 2022-09-10

**Authors:** Leslie Marisol Lugo-Gavidia, Janis M. Nolde, Revathy Carnagarin, Dylan Burger, Justine Chan, Sandi Robinson, Erika Bosio, Vance B. Matthews, Markus P. Schlaich

**Affiliations:** 1 Dobney Hypertension Centre, Medical School—Royal Perth Hospital Unit, Royal Perth Hospital Medical Research Foundation, The University of Western Australia, Perth, WA 6000, Australia; 2 Kidney Research Centre, The Ottawa Hospital Research Institute, Department of Cellular and Molecular Medicine, University of Ottawa, Ottawa, ON K1N 6N5, Canada; 3 Centre for Clinical Research in Emergency Medicine, Harry Perkins Institute of Medical Research, Perth, WA 6150, Australia; 4 Department of Internal Medicine, Royal Perth Hospital, Perth, WA 6000, Australia; 5 Departments of Cardiology and Nephrology, Royal Perth Hospital, Perth, WA 6000, Australia

**Keywords:** extracellular vesicles, risk score, platelets

## Abstract

Elevated circulating platelet-derived extracellular vesicles (EVs) have been reported in conditions associated with thrombotic risk. The present study aimed to assess the relationship between circulating platelet-derived EV levels, cardiovascular risk stratification and vascular organ damage, as assessed by pulse wave velocity (PWV). A total of 92 patients were included in the present analysis. Platelet EV were evaluated by flow cytometry (CD41+/Annexin v+). The cardiovascular risk was determined using the 2021 ESC guideline stratification and SCORE2 and SCORE-OP. PWV was performed as a surrogate to assess macrovascular damage. Risk stratification revealed significant group differences in EV levels (ANOVA, *p* = 0.04). Post hoc analysis demonstrated significantly higher levels of EVs in the very high-risk group compared with the young participants (12.53 ± 8.69 vs. 7.51 ± 4.67 EV/µL, *p* = 0.03). Linear regression models showed SCORE2 and SCORE-OP (*p* = 0.04) was a predictor of EV levels. EVs showed a significant association with macrovascular organ damage measured by PWV (*p* = 0.01). PWV progressively increased with more severe cardiovascular risk (*p* < 0.001) and was also associated with SCORE2 and SCORE-OP (*p* < 0.001). Within the pooled group of subjects with low to moderate risk and young participants (<40 years), those with EV levels in the highest tertile had a trend towards higher nocturnal blood pressure levels, fasting glucose concentration, lipid levels, homocysteine and PWV. Levels of platelet-derived EVs were highest in those patients with very high CV risk. Within a pooled group of patients with low to moderate risk, an unfavourable cardiometabolic profile was present with higher EV levels.

## 1. Introduction

Cardiovascular disease remains a major cause of morbidity and mortality worldwide [1,2]. A combination of various cardiometabolic risk factors paired with poor lifestyle choices and psychosocial stressors represent an important burden on vascular health and contribute to the progression of the disease [2].

International guidelines recommend an initial risk classification to guide therapeutic decision making and tailor interventions at an individual level [2,3,4,5]. To this effect, efforts have been made to develop tools to accurately identify high-risk patients who require more strict risk factor control and therefore more aggressive interventions [2,3,6]. Multiple risk scores have been designed and optimized over the years. Most recently, the Systematic Coronary Risk Estimation 2 (SCORE2) and SCORE-OP (Systematic Coronary Risk Estimation for older persons) were introduced, re-calibrated to estimate both fatal and non-fatal CV events [2]. 

Recently, considerable attention has been devoted to the role of extracellular vesicles as an early biomarker in cardiovascular disease. Large extracellular vesicles (EVs), also termed microparticles, microvesicles or ectosomes, are small cell vesicles derived from the cell membrane of different cells in response to biological processes. The basis of the large EV biogenesis is the outward blebbing of the plasma membrane with the externalization of phosphatidylserine [7]. Previous data showed increased circulating EV levels in the presence of a range of cardiovascular conditions and risk factors such as diabetes, hypertension, obesity, and dyslipidemia [7,8,9,10,11]. Platelet-derived EVs have a strong pro-coagulant property and have been related to inflammation, a key feature of the initiation and progression of atherosclerotic cardiovascular disease and the onset of clinical events [12,13,14,15]. 

Arterial stiffness has been widely recognized as a surrogate for cardiovascular disease, current guidelines recommend the assessment of arterial stiffness to evaluate HMOD and underlying arteriosclerosis, especially in patients who appear to be asymptomatic. Pulse wave velocity (PWV) is a non-invasive bedside test to evaluate arterial stiffness. A PWV > 10 m/s is considered to represent significant alterations in the arterial elastic properties and determine subclinical HMOD [16,17,18,19].

Several studies have investigated the relationship of EV with different cardiovascular risk factors individually. In this analysis, we use the new 2021 ESC risk estimation and SCORE2 and SCORE-OP as an integrated evaluation of cardiovascular risk factors. The present study aimed to evaluate if platelet-derived EV concentrations correlate with the risk stratification determined by the recently published SCORE2 and SCORE-OP, as well as their relationship with macrovascular damage evaluated by PWV. 

## 2. Results

### 2.1. Baseline Characteristics

A total of 92 patients were included in the study. The study cohort consisted of 37 (40.2%) very high-risk, 26 (28.3%) high-risk and 11 (12%) low-to-moderate-risk patients, and 18 (19.6%) were young patients (<40 years old). Baseline demographics and clinical characteristics of the study population according to the risk categories are summarized in Table 1. As expected, several differences were observed according to their cardiovascular risk. The very high-risk population was older and had higher blood pressure, glucose and HbA1c levels. They also exhibited significantly lower eGFR and an increased urinary albumin–creatinine ratio. 

### 2.2. Assessment of Extracelular Vesicles and Cardiovascular Risk

**Figure 1:**
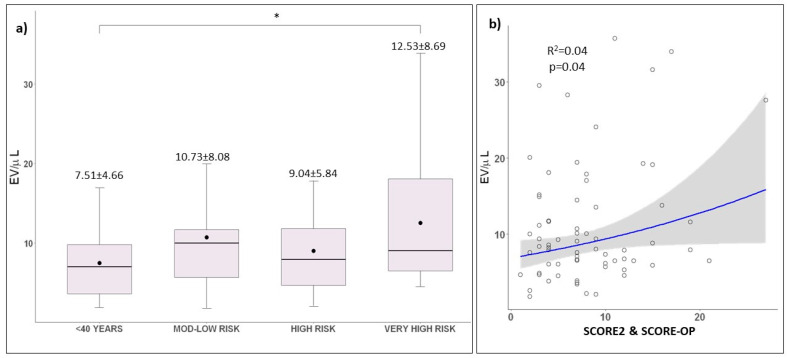
Extracellular vesicles and risk categories. (**a**) Boxplot of risk categories and EV (ANOVA; *p* = 0.04). Black dots represent the mean. Asterisks represent adjusted significant post hoc Tukey test between groups (* *p* < 0.05). Post hoc Tukey pairwise comparations: <40 years vs. low-to-moderate risk: *p* = 0.68, <40 years vs. high risk: *p* = 0.76, <40 years vs. very high risk: *p* = 0.03, low-to-moderate risk vs. high risk: *p* = 0.98, low-to-moderate risk vs. very high risk: *p* = 0.71, high risk vs. very high risk: *p* = 0.22. (**b**) Regression model of extracellular vesicles and SCORE2 and SCORE-OP (*p* = 0.04). Blue line represents regression line of best fit. The grey bands around the line represent 95% confidence intervals.

Higher levels of platelet-derived EVs were observed in the very high-risk group (12.53 ± 8.69 EV/µL) compared to high-risk, low-to-moderate risk and young patients (9.04 ± 5.84 EV/µL, 10.73 ± 8.08 EV/µL and 7.51 ± 4.66 EV/µL, respectively). An analysis of variance (ANOVA) showed a significant difference of EV levels among the risk groups, F(3;8) = 2.89, *p* = 0.04. (Figure 1a). Post hoc Tukey tests for individual group analysis revealed significant differences between very high-risk patients and young participants. (*p* = 0.03). A linear regression model showed that SCORE2 and SCORE-OP was a significant predictor of EV concentrations (F(1;68) = 4.25; *p* = 0.04), with an R^2^ of 0.04 (Figure 1b). 

### 2.3. Target Organ Damage Evaluation

**Figure 2:**
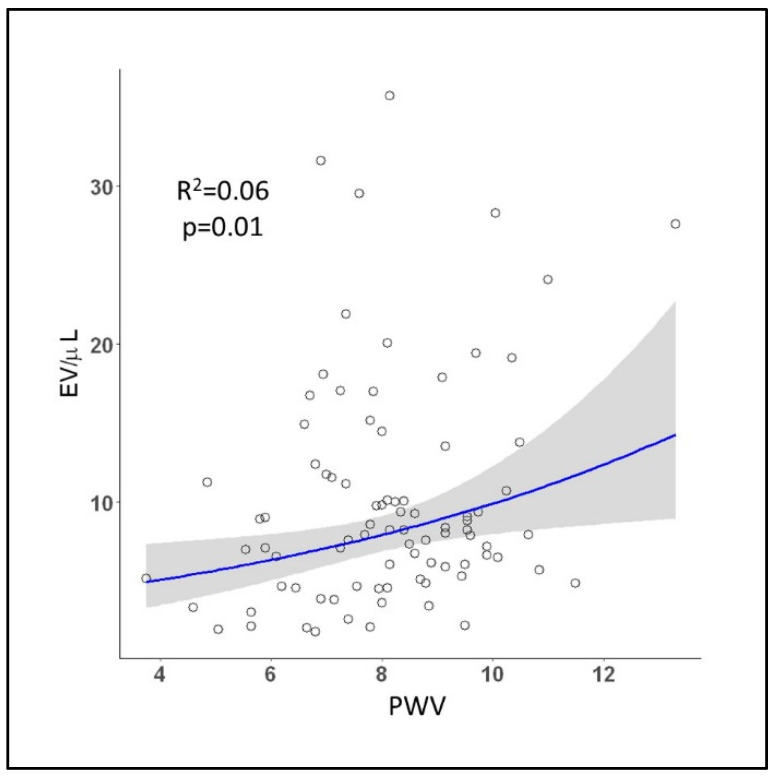
Extracellular vesicles and macrovascular damage. Regression model of extracellular vesicles and PWV (*p* = 0.01). Blue line represents regression line of best fit. The grey bands around the line represent 95% confidence intervals.

**Figure 3:**
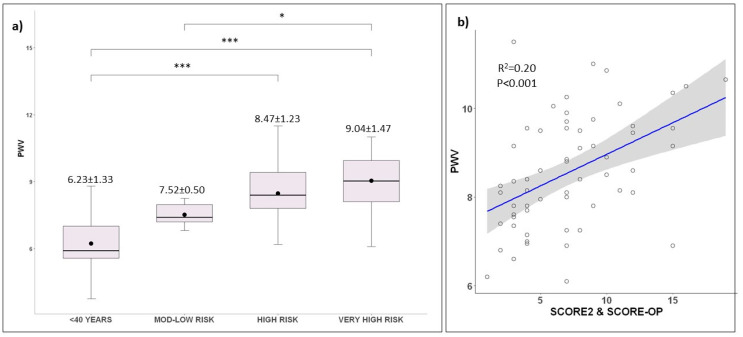
Macrovascular damage and risk categories. (**a**) Boxplot of risk categories and PWV (ANOVA; *p* < 0.001). Black dots represent the mean. Asterisks represent adjusted significant post hoc Tukey test between groups (* *p* < 0.05, *** *p* < 0.001). Post hoc Tukey pairwise comparations: <40 years vs. low-to-moderate risk: *p* = 0.06, <40 years vs. high risk: *p* < 0.001, <40 years vs. very high risk: *p* < 0.001, low-to-moderate risk vs. high risk: *p* = 0.20, low-to-moderate risk vs. very high risk: *p* = 0.01, high risk vs. very high risk: *p* = 0.34. (**b**) Regression model of PWV and SCORE2 and SCORE-OP (*p* < 0.001). Blue line represents regression line of best fit. The grey bands around the line represent 95% confidence intervals.

A regression model showed that EVs were associated with macrovascular organ damage assessed by PWV (R^2^ = 0.06; *p* = 0.01) (Figure 2). There was no significant correlation with other PWA parameters. Group stratification showed a progressive increase in PWV values with more severe cardiovascular risk (*p* < 0.001) (Figure 3a). A regression model showed a significant association between PWV and SCORE2 and SCORE-OP (R^2^ = 0.20; *p* < 0.001) (Figure 3b). PWV prediction by SCORE2 and SCORE-OP was not improved when EVs were added to the model. As expected, young participants showed a significantly lower augmentation index compared to the other groups (Table 1). 

### 2.4. Cardiovascular Risk Groups’ Sub-Analysis

In view of the differences observed between the risk categories, as a sub-analysis, we performed regression models, excluding very high-risk participants. PWV and SCORE associations with EVs were not significant (*p* = 0.12 and *p* = 0.73, respectively). These results reflect the important contribution of very high risk on these parameters and the differences observed in the individual group comparison.

We then pooled the group of young patients and those classified as low-to-moderate-risk and divided the group into tertiles based on EV levels. A numerical but not statistically significant trend toward a greater burden of cardiovascular risk factors (hypertension and dyslipidemia) was found among patients in the upper tertile of EV concentration. Patients in the upper tertile (T3) also showed numerically higher nocturnal blood pressure levels (T3: 119 ± 20.6, T2: 113 ± 12.5, T1: 111 ± 14.6; *p* = 0.60) and a worse nocturnal dipping pattern (T3: 5.39 ± 11.3, T2: 11.7 ± 5.98, T1: 7.83 ± 9.28; *p* = 0.43). Interestingly, there was a higher proportion of patients presenting white coat hypertension in the lower tertile (*p* < 0.001). Patients presenting masked hypertension were more frequent in the upper tertile. Their pathology results showed a numerical trend to higher levels of glucose, HbA1c, insulin resistance score (HOMA-IR), lipid levels and homocysteine (Table 2). Finally, patients in the upper tertile also showed numerically higher levels of PWV and AIx (Table 2). 

## 3. Discussion

Our study has several key findings: (1) EVs were significantly associated with 10-year risk estimation and PWV; (2) CV risk stratification showed a progressive increase in PWV values with higher-risk categories and the 10-year risk of fatal and non-fatal CV events (SCORE2 and SCORE-OP) was significantly correlated with more pronounced macrovascular damage; (3) When analyzing low-to-moderate-risk patients and young patients (<40) together, we found that participants in the upper EV tertile showed a trend towards an unfavourable cardiometabolic profile.

The risk estimation system measures established risk factors at a single time point; it allows us to identify patients at high risk who would require more immediate and more intense interventions. While risk stratification evaluates the integrated cardiovascular risk, it may not reflect the cumulative exposure over time. Patients with no risk factors or only mildly elevated risk are not generally included in clinical trials, and the international guidelines suggest a less intensive approach. Commonly, a stepwise therapy escalation is suggested with young and low-to-moderate-risk patients, as the risk of CV events is not immediately perceived. The previous might be true in the large majority; however, recent data have suggested that a group of patients can develop major adverse events in the absence of standard modifiable risk factors [20,21,22,23,24] and is associated with adverse outcomes [22,23]. In addition, it has been reported that only a low proportion of these patients met the criteria for starting therapy (e.g., statins) before a CV event [25]. These data illustrate the urgent need for finding new tools that help to identify subclinical disease, irrespective of a perceived low risk and the importance of early prevention strategies. It is important to emphasize that risk estimations are not calibrated, therefore not recommended for young people (<40 years old). Importantly, patients presenting mildly elevated risk factors at a young age have a longer exposure that translates to a higher disease burden and might act as an amplifier of further risk.

In our study, we observed a progressive increase in PWV values with increased cardiovascular risk. This observation is consistent with the literature [18,26]. Currently, PWV is suggested as a non-invasive method to measure arterial stiffness, which is considered a surrogate for subclinical target organ damage and has been suggested as a tool to predict CV risk and improve risk stratification [18,26,27,28,29]. A meta-analysis conducted by Vlachopoulos et al. showed a linear association of PWV with clinical events. Overall, the RRs for 1m/s increase in PWV were 1.14 [95% CI 1.09–1.20], *p* < 0.001, for total clinical events and 1.15 [95% CI 1.09–1.21], *p* < 0.001, for cardiovascular mortality [18]. Furthermore, in the EDIVA (Estudo de DIstensibilidade VAscular) project [26], PWV was incorporated into the HeartSCORE and categorized in low-, intermediate- and high-risk patients; the event-free survival at 2 years was 98.6%, 98.0% and 96.1%, respectively. The study showed an improvement in risk classification when PWV was added to the model [26], highlighting the potential utility of incorporating other parameters into risk assessment, particularly in patients with low-to-intermediate risk for improved CV risk classification [6,26].

EVs are small plasma membrane vesicles released from different cells in response to biological processes. Platelet-derived EV levels have been associated with several cardiovascular risk factors and have been suggested as a potential marker for thrombosis, as they express important procoagulant capacity [12,15,30,31,32]. Our data indicate that the total risk SCORE can predict levels of EV, suggesting that CV risk contributes, at least partially, to EV biogenesis. Furthermore, there was a significant difference when comparing risk categories, with higher levels of platelet-EVs significantly associated with a very high risk. A previous study showed higher levels of endothelial EVs were associated with an increased Framingham score [33]. In our study, we incorporate the new and updated SCORE2 algorithm that accounts for both fatal and non-fatal cardiovascular events, which prevents the underestimation of total cardiovascular burden, especially in younger patients that are prone to present non-fatal events [6]. We also evaluate cardiometabolic parameters in those patients presenting low-to-moderate risk and young participants according to EV tertiles. Interestingly, patients in the upper tertile showed a trend toward a higher cardiovascular burden (higher proportion of hypertension and dyslipidemia). Of note, the different hypertensive phenotypes (white coat hypertension and masked hypertension) had an interesting distribution among the EV tertiles. While masked hypertension was only present in T2 and T3, the white coat phenotype, which in general is characterized by less pronounced or no organ damage compared to sustained hypertension, was significantly more prevalent in the lower tertile. Higher levels of common cardiometabolic risk parameters, including glucose levels and aspects of the lipid profile, were observed in the upper tertile. Although these results did not reach statistical significance (possibly due to the small sample size), they may have clinical relevance and perhaps suggest that EVs start to increase even in the early stages of the disease process. Additionally, the fact that PWV was significantly correlated with EV and that the upper tertile of EVs also expressed higher levels of PWV and Aix indicate that EVs possibly reflect vascular changes. 

This study has some limitations. First, given the cross-sectional nature of this analysis, it is limited to inferring causal relationships. For patients referred and treated previously by other physicians, we use their status at the time of inclusion in the study. The duration of the disease and treatments before the visit are not considered in the present analysis. Consequently, our results represent the current state of risk and are limited to evaluating the effect of long-standing disease on more severe vascular damage. Secondly, a small proportion of baseline data of some participants was not available at the time of analysis. However, as risk categories represent an integrated cardiovascular evaluation, this is unlikely to impact the general interpretation of the results. Finally, the small sample size of the low-to-moderate group might have limited the capacity to evaluate significant differences between EV tertiles; hence, these results must be considered only hypothesis-generating. We acknowledge the inherent limitations of the present investigation due to its observational nature, use of PWV as a surrogate of macrovascular damage and a small sample size for low-to-moderate-risk patients. 

## 4. Materials and Methods

### 4.1. Subject Population and Study Design 

The study included a total of 92 patients between 18 and 85 years old attending for diagnostic, workup and clinical management of hypertension and cardiovascular disease at the outpatient clinic of Royal Perth Hospital. The patients were stratified into four different categories according to their cardiovascular risk following the 2021 ESC guidelines on cardiovascular disease prevention in clinical practice [2]. In summary, patients with established atherosclerotic cardiovascular disease (ASCVD), patients with type 2 diabetes mellitus (DM), patients with target organ damage (TOD) and patients with severe chronic kidney disease (CKD) (eGFR < 30 mL/min/1.73 m^2^ or eGFR 30–40 mL/min/1.73 m^2^ and albumin–creatinine ratio > 3 mg/mmol) were classified as *very high risk*. ASCVD was defined as a history of acute myocardial infarction, acute coronary syndrome, any arterial revascularization, stroke, transitory ischaemic attack, peripheral artery disease, or documented ASCVD. Patients with moderate CKD and patients with long-standing diabetes or uncontrolled diabetes without TOD were classified as *high risk*. Patients presenting with office blood pressure values > 180 mmHg were also classified as *high risk*. In people without ASCVD, DM, CKD, or familiar hypercholesterolemia and an age between 40 and 89 years, we used the SCORE2 and SCORE-OP to estimate the 10-year CV risk and categorized them as *very high risk*, *high risk*, or *low to moderate risk* in line with age-adjusted risk thresholds (Supplementary Materials online, Table S1) [2]. 

Finally, we included an additional category of patients without ASCVD, DM, CKD, and <40 years old in whom the SCORE2 and SCORE-OP are not applicable to estimate risk due to very low perceived risk.

The study complied with the Declaration of Helsinki and received approval from the University of Western Australia research ethics committee. All participants provided written consent for the study. Clinical baseline data were collected from the patients, including medical history, medication history, serum pathology and blood pressure evaluation.

### 4.2. Platelet EV Characterization

Platelet EV subpopulations were evaluated by flow cytometry according to the expression of platelet markers (CD41), as described previously by our group [34]. Briefly, venous blood was collected after 10–12 h of fasting into 3.8% sodium citrate tubes. The first 3 mL of blood was discarded, to avoid platelet activation. Platelet-free plasma (PFP) was obtained by successive centrifugations at 800× *g* for 10min and double centrifugation at 2500× *g* for 15 min at room temperature (RT). PFP was immediately frozen and stored at −80 °C until processing for isolation and quantification. All samples were processed identically and within 1 hr after extraction. Samples that failed to accurately measure EVs (e.g., insufficient volume or hemolysis) were excluded from the analysis.

To isolate large EVs, PFP frozen aliquots were thawed at RT and centrifuged at 12,000× *g* for 2 min to remove fibrin clots/aggregates. The supernatant (400 μL) was collected for subsequent high-speed centrifugation at 20,000× *g* for 20 min. The supernatant was discarded, and the remaining EV-enriched pellet was re-suspended in 300 μL ultrafiltered PBS. Re-suspended EVs were incubated for 60 min with fluorochrome-labelled antibodies (CD41-PE-Cy7). The mix was subsequently incubated with Annexin V-FITC at 5% for 10 min and diluted with ultra-filtered annexin binding buffer (10 mM HEPES, pH 7.4, 140 mM NaCl, 2.5 mM CaCl2), before being immediately analyzed on an Attune^TM^ NxT Acoustic Focusing Cytometer. Equivalent concentrations of the respective isotype controls were used to determine the degree of non-specific binding. The acquisition was performed using the lower flow rate (12.5 μL/min). Forward scatter (FSC), side scatters (SSC) and fluorescence data were obtained with the settings in the logarithmic scale. The concentration of EVs was determined by volumetric cell count in 50 µL of the sample within gate limits established by ApogeeMix (Apogee Flow Systems). The lower detection threshold was set using the 80 nm fluorescent/180 nm silica beads signal. EVs within the established gate limits were identified and quantified based on their binding to Annexin V and reactivity to CD41-PECy7 to define platelet-derived EVs (pEVs) (Supplementary Materials online, Figure S1).

### 4.3. Markers of Macrovascular Organ Damage

Arterial stiffness was assessed by non-invasive pulse wave analysis (PWA) and pulse wave velocity (PWV) with the SphygmoCor XCEL system (AtCor Medical Pty Ltd., Australia), as per the manufacturer’s recommendations. PWA was performed after a 5 min rest period in the supine position; an automatic 10s PWA reading was used for acquiring the hemodynamic parameters (central mean arterial pressure (cMAP), aortic augmentation pressure (AP) and augmentation index (AIx)). Simultaneous measurements through applanation tonometry over the carotid and femoral artery provide the pulse transit time. PWV assessment was set to 10 s with a PWV distance and subtraction method and expressed as distance/transit time (m/s). PWV assessments were performed twice; only measurements achieving the internal quality control both times were considered valid and the average was used for further analysis. 

### 4.4. Statistical Analysis

For baseline characteristics, continuous variables were expressed as mean ± SD and categorical variables as frequencies and percentages. Qualitative variables were compared with the chi-square test or Fisher’s exact test if application conditions were not fulfilled. The Pearson correlation coefficient was used for correlation analyses for continuous variables. A linear regression model was fit to test if the absolute SCORE2 and SCORE-OP as an integrated evaluation of cardiovascular risk factors predict EV levels. The models were not adjusted by any other variable, as the risk categories and SCORE2 and SCORE-OP represent an integrated value of the variables known to affect PWV, PWA and EV. Non-parametric tests were applied when required. Normality of the data was assessed by graphical methods Q–Q plots and the Shapiro–Wilk test. Log transformation of EV was applied to the analysis to achieve a normal distribution of the models (Shapiro–Wilk normality test *p* = 0.32). Graphical representations display the fitted model accounting for transformations applied and are presented in the original scale (EV/µL) to allow understanding of the EV level’s behavior. Differences in quantitative variables between risk categories were made with the one-way ANOVA method (Bartlett’s Test for Homogeneity of Variances, *p* = 0.56). A post hoc Tukey test was performed to evaluate all between-group and within-group comparations (EV tertiles). A *p*-value < 0.05 was considered statistically significant for all comparisons. Statistical analysis was performed using R 4.0.3 software. 

## 5. Conclusions and Perspectives

In conclusion, our results demonstrate a statistically significant correlation between the new SCORE2 and SCORE-OP and CV risk. Higher levels of EVs were associated with very high-risk patients. EVs were also associated with macrovascular damage evaluated by PWV. In low-to-moderate-risk patients, patients in the upper EV tertile had a tendency to have higher levels of cardiometabolic parameters. While our correlations were not very strong, the importance of our results remains as proof of concept of the contribution of CV risk on the release of EVs. Although it is generally recognized that cardiovascular diseases are associated with higher levels of EVs, the exact mechanisms of their release have still not been fully elucidated. The literature has reported significant associations of EV with multiple conditions (e.g., thrombosis, inflammation); it becomes interesting to hypothesize that EV generation is influenced by multiple factors. While its use in clinical practice is still not clear, it is a growing field, as they have been proven to participate as mediators of intercellular communication, regulate physiological responses and influence miRNA expression, among others. It is therefore an interesting option to understand the underlying mechanisms of cardiovascular disease. Larger longitudinal studies including hard outcomes are required to determine to what extent the incorporation of EVs to risk evaluation increases the discriminative power for major cardiovascular events.

## Figures and Tables

**Table 1:** Baseline characteristics stratified by risk categories.

	Overall	<40 Years	Mod-Low Risk	High Risk	Very High Risk	*p*-Value
	(N = 92)	(N = 18)	(N = 11)	(N = 26)	(N = 37)	
Male	54 (58.7%)	9 (50.0%)	2 (18.2%)	19 (73.1%)	24 (64.9%)	0.01
Age	55.5 ± 15.1	32.4 ± 5.75	54.8 ± 5.09	54.9 ± 10.1	67.4 ± 8.13	<0.001
BMI (Kg/m^2^)	30.1 ± 5.97	25.3 ± 5.89	31.3 ± 7.37	32.0 ± 5.18	30.6 ± 4.98	0.001
Diabetes	25 (27.2%)	-	-	8 (30.8%)	17 (45.9%)	<0.001
Hypertension	81 (88.0%)	8 (44.4%)	11 (100%)	25 (96.2%)	37 (100%)	<0.001
Dyslipidemia	58 (63.0%)	2 (11.1%)	7 (63.6%)	16 (61.5%)	33 (89.2%)	<0.001
Coronary artery disease	13 (14.1%)	-	-	-	13 (35.1%)	-
Stroke/TIA	4 (4.3%)	-	-	-	4 (10.8%)	-
Peripheral artery disease	4 (4.3%)	-	-	-	4 (10.8%)	-
Glucose (µmmol/L)	5.98 ± 1.67	5.01 ± 0.448	5.20 ± 0.673	6.04 ± 1.60	6.48 ± 1.98	0.03
HbA1c (%)	6.18 ± 1.42	5.19 ± 0.285	5.55 ± 0.459	6.33 ± 1.27	6.63 ± 1.70	0.03
Total cholesterol (mmol/L)	4.89 ± 1.13	4.64 ± 1.13	5.47 ± 0.822	5.24 ± 0.972	4.55 ± 1.22	0.02
Triglyceride (mmol/L)	1.60 ± 1.04	1.29 ± 1.38	1.44 ± 0.741	1.82 ± 1.19	1.61 ± 0.868	0.47
HDL cholesterol (mmol/L)	1.30 ± 0.391	1.30 ± 0.333	1.58 ± 0.610	1.24 ± 0.318	1.26 ± 0.352	0.08
LDL cholesterol (mmol/L)	2.87 ± 0.921	2.79 ± 0.697	3.24 ± 0.661	3.20 ± 0.906	2.56 ± 0.973	0.02
Creatinine (µmol/L)	77.0 ± 27.6	78.8 ± 18.2	65.6 ± 7.26	71.1 ± 14.9	83.8 ± 37.7	0.14
eGFR (mL/min/1.73 m^2^)	84.4 ± 11.2	88.5 ± 4.50	89.0 ± 1.76	87.8 ± 5.66	77.9 ± 15.4	0.001
UACR (mg/mmol)	2.07 ± 3.97	1.66 ± 3.21	0.88 ± 0.504	1.10 ± 1.07	3.50 ± 5.86	0.23
Sys AOBP (mmHg)	132 ± 18.6	119 ± 13.1	127 ± 18.2	134 ± 13.8	139 ± 20.6	0.001
Dia AOBP (mmHg)	79.1 ± 13.6	74.6 ± 11.3	83.0 ± 15.3	84.2 ± 10.9	76.5 ± 14.7	0.04
PWV (mmHg)	8.11 ± 1.67	6.23 ± 1.33	7.52 ± 0.501	8.47 ± 1.23	9.04 ± 1.47	<0.001
Central MAP (mmHg)	96.3 ± 12.7	87.4 ± 10.3	101 ± 19.6	100 ± 8.72	96.7 ± 12.1	0.004
Augmentation index (mmHg)	19.0 ± 13.6	3.88 ± 11.7	25.9 ± 10.6	22.1 ± 11.7	22.7 ± 10.9	<0.001

*p*-values refer to ANOVA between groups. Data are shown as mean and standard deviation for continuous variables and frequencies and percentages for categorical variables. AOBP: Automated office blood pressure, UACR: urine albumin–creatinine ratio, PWV: pulse wave velocity, MAP: Mean arterial pressure.

**Table 2:** Clinical characteristics of participants with low-to-moderate CV risk stratified by platelet EV tertiles.

	T1	T2	T3	*p*-Value *
	(N = 10)	(N = 10)	(N = 9)	
Dyslipidemia	2 (20.0%)	2 (20.0%)	5 (55.6%)	0.18
Hypertension	5 (50.0%)	6 (60.0%)	8 (88.8%)	0.23
Age	39.3 ± 12.2	36.7 ± 11.7	47.3 ± 11.8	0.16
BMI (kg/m^2^)	27.0 ± 8.24	27.5 ± 7.16	28.2 ± 6.16	0.94
White cell count (10^9^/L)	5.26 ± 0.973	5.69 ± 1.87	5.68 ± 1.43	0.83
Red cell count (10^9^/L)	4.59 ± 0.386	4.78 ± 0.407	4.55 ± 0.272	0.51
Haematocrit (L/L)	0.410 ± 0.0245	0.423 ± 0.0207	0.413 ± 0.0287	0.62
Haemoglobin (g/L)	137 ± 14.5	141 ± 7.37	138 ± 9.97	0.86
Platelet count (10*9/L)	244 ± 80.6	249 ± 49.7	220 ± 23.5	0.62
Glucose (mmol/L)	5.10 ± 0.454	4.93 ± 0.486	5.30 ± 0.772	0.52
HOMA-IR	1.66 ± 1.16	1.87 ± 1.38	2.43 ± 1.15	0.61
HbA1c (%)	5.40 ± 0.381	5.07 ± 0.327	5.56 ± 0.336	0.09 ^¥^
Total cholesterol (mmol/L)	4.83 ± 0.814	4.71 ± 0.866	5.53 ± 1.38	0.27
Triglycerides (mmol/L)	1.04 ± 0.431	1.46 ± 0.956	1.58 ± 1.67	0.62
HDL cholesterol (mmol/L)	1.53 ± 0.427	1.34 ± 0.355	1.43 ± 0.680	0.79
LDL cholesterol (mmol/L)	2.83 ± 0.483	2.74 ± 0.586	3.41 ± 0.853	0.12
eGFR (mL/min/1.73 m^2^)	87.1 ± 5.67	89.2 ± 1.33	89.9 ± 0.7	0.3
Homocysteine (µmol/L)	9.50 ± 2.42	8.30 ± 0.548	10.1 ± 2.67	0.52
Systolic AOBP (mmHg)	123 ± 19.0	124 ± 10.3	120 ± 17.4	0.88
Diastolic AOBP (mmHg)	81.9 ± 18.0	73.8 ± 11.0	77.8 ± 9.00	0.41
ABPM 24h—SBP (mmHg)	119 ± 11.8	125 ± 12.5	124 ± 15.5	0.62
ABPM 24h—DBP (mmHg)	75.6 ± 11.6	71.1 ± 9.97	78.8 ± 9.57	0.39
ABPM Day—SBP (mmHg)	121 ± 12.3	128 ± 13.3	126 ± 15.3	0.57
ABPM Day—DBP (mmHg)	77.4 ± 11.9	73.6 ± 10.1	80.8 ± 10.7	0.46
ABPM Night—SBP (mmHg)	111 ± 14.6	113 ± 12.5	119 ± 20.6	0.60
ABPM Night—DBP (mmHg)	69.0 ± 16.3	61.0 ± 9.81	72.4 ± 11.0	0.23
Nocturnal systolic dipping (%)	7.83 ± 9.28	11.7 ± 5.98	5.39 ± 11.3	0.43
Nocturnal diastolic dipping (%)	10.9 ± 15.2	17.1 ± 6.21	9.93 ± 11.4	0.45
White coat hypertension phenotype	2 (20.0%)	1 (10.0%)	0 (0.0%)	<0.001
Masked hypertension phenotype	0 (0.0%)	3 (30.0%)	2 (22.2%)	0.23
PWV (m/s)	6.42 ± 1.24	6.66 ± 1.50	7.07 ± 1.01	0.57
Mean arterial pressure (mmHg)	94.6 ± 21.9	91.0 ± 13.0	90.9 ± 8.51	0.85
Augmentation index (%)	11.8 ± 17.6	3.67 ± 14.1	20.0 ± 10.3	0.09 ^¥^

* *p*-values for ANOVA, ^¥^ *p*-values of T2 vs. T3 showed significant differences (*p* < 0.05) in the adjusted post hoc Tukey test between groups. Data are shown as mean and standard deviation for continuous variables and frequencies and percentages for categorical variables. HOMA-IR: Homeostatic model assessment insulin resistance, PWV: pulse wave velocity, AOBP: Automated office blood pressure, UACR: urine albumin–creatinine ratio, T1: lower tertile, T2: mid tertile, T3: upper tertile.

## Data Availability

Data cannot be shared.

## Supplementary Materials

The supporting information can be downloaded at: https://www.mdpi.com/article/10.3390/ijms231810524/s1.
