# Supplementary material for: Association of Circulating Platelet Extracellular Vesicles and Pulse Wave Velocity with Cardiovascular Risk Estimation
Source: Int J Mol Sci. 2022 Sep 10;23(18):10524. doi: 10.3390/ijms231810524 (PMC9505165; doi:10.3390/ijms231810524)
Supplement: Supplementary file 1 [file ijms-23-10524-s001.zip › ijms-1895327-supplementary.pdf]

Supplementary material

Table S1. Cardiovascular risk categories according to age thresholds

	<50 years	50-69 years	≥70 years
Low to moderate CVD risk	<2.5%	<5%	<7.5%
High CVD risk	2.5 to <7.5%	5 to <10%	7.5 to <15%
Very high CVD risk	≥7.5%	≥10%	≥15%

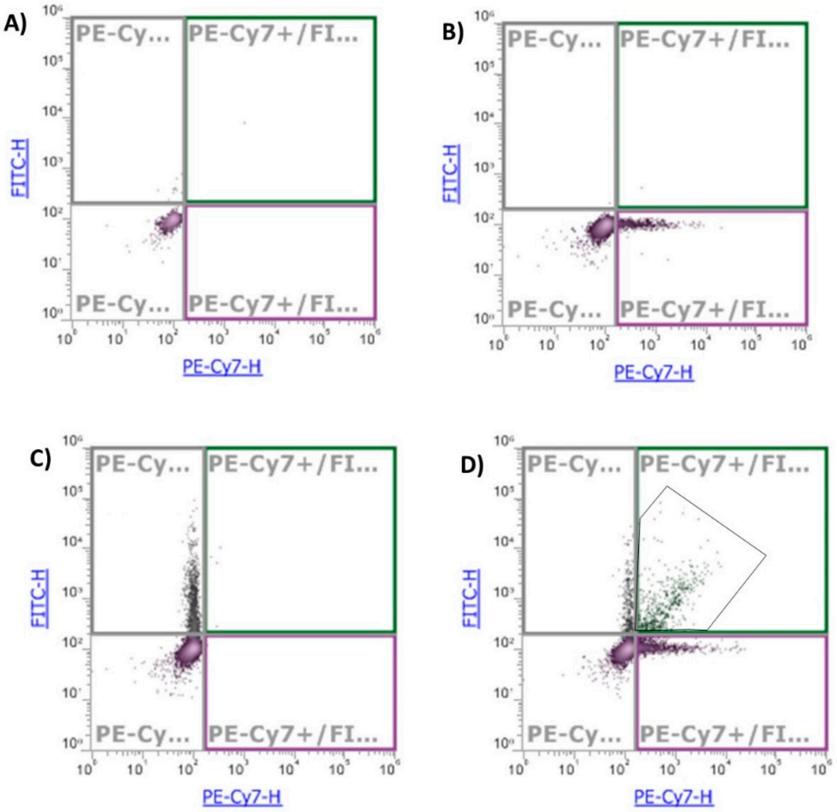

Figure S1. Gating and acquisition strategy for the detection of circulating extracellular vesicles with flow cytometry. A) Buffer only. **B)** CD41-PE Cy7 single stain. **C)** Annexin V-FITC single stain. **D)** Double staining with CD41-PE Cy7 and Annexin V-FITC.
